# Simultaneous Quantification of Mitochondrial DNA Damage and Copy Number in Circulating Blood: A Sensitive Approach to Systemic Oxidative Stress

**DOI:** 10.1155/2013/157547

**Published:** 2012-12-27

**Authors:** Sam W. Chan, Simone Chevalier, Armen Aprikian, Junjian Z. Chen

**Affiliations:** Department of Surgery/Division of Urology, The Research Institute of McGill University Health Center, 1650 Cedar Avenue, Room R1.107, Montreal, QC, Canada H3G 1A4

## Abstract

Systemic oxidative stress is associated with a wide range of pathological conditions. Oxidative DNA damage is frequently measured in circulating lymphocytes. Mitochondrial DNA (mtDNA) is known to be more sensitive to oxidative damage than nuclear DNA but is rarely used for direct measurement of DNA damage in clinical studies. Based on the supercoiling-sensitive real-time PCR method, we propose a new approach for the noninvasive monitoring of systemic oxidative stress by quantifying the mtDNA structural damage and copy number change in isolated lymphocytes in a single test. We show that lymphocytes have significantly less mtDNA content and relatively lower baseline levels of damage than cancer cell lines. In an *ex vivo* challenge experiment, we demonstrate, for the first time, that exogenous H_2_O_2_ induces a significant increase in mtDNA damage in lymphocytes from healthy individuals, but no repair activity is observed after 1 h recovery. We further demonstrate that whole blood may serve as a convenient alternative to the isolated lymphocytes in mtDNA analysis. Thus, the blood analysis with the multiple mtDNA end-points proposed in the current study may provide a simple and sensitive test to interrogate the nature and extent of systemic oxidative stress for a broad spectrum of clinical investigations.

## 1. Introduction

Oxidative stress is a state of physiological imbalance between oxidant production and antioxidant defence at different biological levels. It is implicated in the development of many pathological conditions such as aging, neurodegenerative diseases, and cancer initiation and progression [1–6]. Many diseases are suspected to be linked to oxidative stress, but procurement of disease tissues may be difficult due to its invasive nature and the scarcity of available tissues. However, researchers have mitigated this problem by using the systemic oxidative stress in peripheral tissues, such as circulating blood, as a noninvasive surrogate. Extrinsic factors such as inflammation, nutrient imbalance, and hypoxic environment could affect inter and intracellular redox homeostasis, therefore altering systemic oxidative stress levels; new efforts are made to investigate the interactions between systemic oxidative stress and pathogenesis of many disease conditions [7–13]. For example, several recent studies suggest a correlation between increased systemic oxidative stress and prostate cancer risk and progression [14–16]. Similar results are reported in lung cancer [17], head and neck cancer [18], and other human cancers [19, 20]. Thus, enhanced oxidative stress is not only a common property of the diseased cells, but may also be reflected in the peripheral tissues.

Systemic oxidative stress has been analyzed in serum and blood cells using different biomarkers and assay systems. Genomic DNA in circulating lymphocytes is a widely used target in measuring different end-points of oxidative DNA damage, such as 8-oxoguanine (8-oxo-G) base lesions or DNA strand breaks detected with the comet assay [14–18]. The mitochondrial DNA (mtDNA) in lymphocytes is an attractive alternative target to determine systemic oxidative stress. MtDNA is a circular, multicopy cytoplasmic DNA, semiautonomously maintained in mitochondria. It is known to be more sensitive to oxidative damage than nuclear DNA [21–23] and has been increasingly used for evaluating systemic oxidative stress with various assays. Similarly to nuclear DNA, 8-oxo-G base lesions can be assessed in purified mtDNA from lymphocytes [24]. Extracellular circulating mtDNA in serum is another marker recently used for evaluating genetic integrity and cancer risk. Elevated levels of free floating mtDNA detected in the plasma or serum are found to be associated with poor prognoses for prostate and testicular germ cell cancers [25–27]. However, the source and nature of this circulating mtDNA are not fully elucidated. Oxidative stress can also affect the total mtDNA content in lymphocytes under various diseased conditions [28]. For example, significant alterations in mtDNA content were detected in lymphocytes from patients with renal cell carcinoma, hyperlipidemia, and Huntington's disease when compared to control populations [19, 29, 30]. However, the relationship between different mtDNA end-points reported in lymphocytes is not clear and the direct measurement of mtDNA strand breaks in lymphocytes has not been reported. We previously developed a sensitive *in vitro* assay to quantify mtDNA structural damage induced by strand breaks, repair and copy number change in prostate cancer cell lines using a supercoiling-sensitive real-time PCR (ss-qPCR) [6, 31]. We showed that oxidative damage can induce single- or double-strand breaks (SSB or DSB), which lead to the disruption of the supercoiled conformation, and that the resulting relaxed conformation is a better qPCR substrate for significantly increased amplification than the supercoiled conformation, even if the starting mtDNA molecules remain the same [31]. Additionally, we observed that prolonged exposure to 95°C heat also introduced strand breaks in the mtDNA. This particular property was advantageously used to disrupt all structural features of mtDNA for precise quantification of the total mtDNA content [31].

The objectives of this current study were to test if the ss-qPCR method could be applied to the lymphocytes and to explore a quantitative strategy to measure multiple mtDNA end-points in circulating blood cells for the study of systemic oxidative stress. We developed an absolute quantification method for precise measurement of mtDNA structural damage, copy number change, and repair activity in blood cells. We demonstrated that mtDNA has low levels of both copy number and baseline damage in lymphocytes as compared to cancer cell lines, and that exogenous H_2_O_2_ led to a significant increase in mtDNA damage but with little repair activity in inactivated lymphocytes in *ex vivo* experiments.

## 2. Materials and Methods

### 2.1. Chemicals, Reagents, and Cell Culture

All chemicals were purchased from Sigma-Aldrich (Oakville, ON, Canada) unless otherwise specified. Prostate cancer cell line LNCaP was purchased from ATCC (Manassas, VA). C4-2, a gift from Dr. L.W.K. Chung, is an isogenic clone of the LNCaP cell line with increased invasive potential [32]. LNCaP and C4-2 prostate cancer cells were cultured in RPMI 1640 complete medium (Invitrogen, Burlington, Ontario, Canada) supplemented with 10% fetal bovine serum (FBS) (Invitrogen) and 1% penicillin-streptomycin (Invitrogen). LNCaP cells were cultured in Poly-L-Lysine (1%) coated dishes. The cells were collected with a Trypsin/EDTA solution, (0.05% trypsin + 0.02% EDTA) then washed down with PBS and stored at −80°C. 

### 2.2. Blood Collection and Lymphocyte Preparation

Healthy male volunteers ranging from 28 to 45 years old were recruited for this pilot study through an institutional review board (REB) approved protocol at the McGill University Health Center. Blood (10 to 15 mL) was collected into 9 mL collection tubes coated with EDTA (Vacu K3EDTA PULL LAV) (Fisher, Monroe, NC). For experiments with whole blood, the samples were immediately stored at −80°C in 10% dimethyl sulfoxide (DMSO) prior to analysis. For experiments with isolated lymphocytes, blood was submitted to Ficoll-Paque Plus (GE Healthcare, Buckinghamshire, England) to recover the lymphocytes [33], then stored at −80°C in 40% RPMI media 1640 supplemented with 50% FBS and 10% DMSO prior to analysis. As per manufacturer's specifications, the extracted sample is composed in majority of lymphocytes (75–93%), with a remaining fraction of monocytes (7–25%) and minimal contaminants from granulocytes, erythrocytes, and platelets (3 ± 2%, 5 ± 2%, and < 0.5%, resp.).

### 2.3. H_2_O_2_ Challenge Experiments with Lymphocytes and Whole Blood

Frozen lymphocytes were thawed in a 37°C water bath for 1-2 min and washed with 5 volumes of ice-cold wash medium (50% FBS and 50% RPMI 1640). The lymphocytes were counted and cell viability was assessed under microscope using the trypan blue dye (average of over 90% viability). A total of *∼*3 × 10^6^ lymphocytes were incubated in 50 mL conical tubes with RPMI-1640 complete medium for 30 min prior to the experiment. The cell suspension was split into three groups of *∼*1 × 10^6^ cells each, treated with 0 (control) or 120 *μ*M H_2_O_2_ for 15 min for exposure or allowed to recover in fresh medium for 60 min. The concentration of 120 *μ*M H_2_O_2_ was chosen to be in the lower-middle range of concentrations used in similar treatments in the literature (50 to 500 *μ*M) [14, 34]. Afterwards, the lymphocyte samples were washed with PBS, spun down to a pellet, and then stored at −80°C before DNA preparation. 

Frozen whole blood was thawed in a 37°C water bath for 1-2 min and washed with 5 volumes of ice-cold PBS wash medium. The whole blood cells were counted with trypan blue dye prior to incubation (average of over 90% viability). A total of *∼*15 × 10^6^ whole blood cells were incubated in RPMI-1640 complete medium in 50 mL conical tubes for 30 min prior to the experiment. Whole blood samples were separated into three groups with *∼*5 × 10^6^ cells each and treated with 0 or 120 *μ*M H_2_O_2_ as in the lymphocyte experiment. Whole blood samples were collected after treatment and stored at −80°C before DNA preparation.

### 2.4. DNA Preparation for the ss-qPCR Analysis

DNA was extracted with the QIAGEN Blood & Cell Culture DNA Kit according to the manufacturer's instructions with minor modifications to ensure that both mtDNA and nuclear DNA were collected together [31, 35]. Total DNA was quantified with a NanoDrop spectrophotometer. DNA template solutions of 1 ng/*μ*L were prepared for each sample with 1X Tris/EDTA Buffer Solution (pH 8.0). Each template solution was split into two equal parts with half serving as an original template for the measurement of the damaged/relaxed mtDNA fraction and the other half heat-treated (95°C for 6 min on a PCR machine) to quantify total amount of mtDNA [31].

### 2.5. Nuclear DNA and mtDNA Standards Preparation for Absolute Quantification

**Figure 1:**
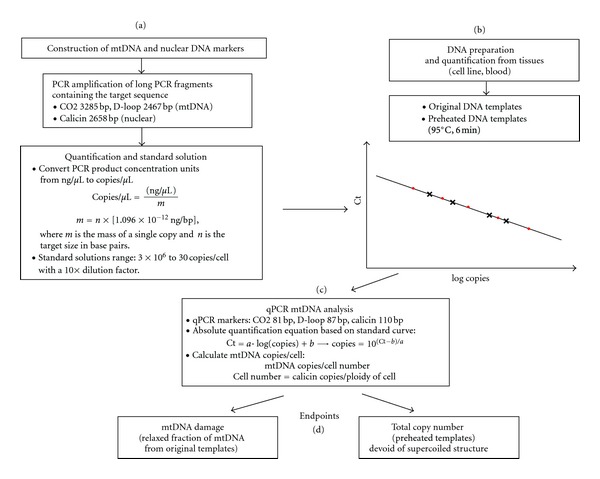
A new strategy for absolute quantification of total and damaged mtDNA. This protocol is separated into four main steps. (a) Construction of the mtDNA and nuclear DNA standards. Long fragments of genes that contained the shorter real-time PCR targets were amplified and a 10X serial dilution was made from 3 × 10^6^ to 30 copies. (b) Preparation of the original and preheated DNA templates from lymphocytes DNA samples for the analysis of mtDNA damage and total mtDNA, respectively. (c) Real-time PCR absolute quantification analysis of mtDNA damage and total content. (d) Interpretation of the data.

MtDNA standards were prepared for absolute quantification. A 3.3 kb mtDNA fragment containing the CO2 gene and a 2.5 kb fragment containing the D-loop region were amplified from the immortalized normal human prostate cell line, RWPE-1, using primers listed in Table 1. PCR reactions were performed using the GeneAmp PCR 9700 system (ABI) with recombinant *Thermus thermophilus* (rTth) DNA polymerase (ABI). The amplification program was performed as follows: preheat samples to reach 75°C; add rTth DNA polymerase and incubate for 2 min; denature at 94°C for 1 min, followed by 30 cycles of 94°C for 15 sec, 60°C for 30 sec., and 72°C for 3.5 min; then 72°C for 5 min and cool down to 10°C. Amplified DNA fragments were purified with the QIAGEN PCR Purification Kit. The purified products were carefully quantified with the Nanodrop spectrophotometer, and the average of three readings was used for calculating precise copy number according to the following equation (Figure 1):

(1) $$\begin{array}{c} \text{copies}/\mu \text{L}=\frac{\left[\text{ng}/\mu \text{L}\right]}{\text{m}}. \end{array}$$

Six or seven serial dilutions were made ranging from 3 × 10^6^ to 30 or 3 × 10^7^ to 30 copies with a dilution factor of 10 depending on the experiment. The 6-point standard was used for the mtDNA quantification in blood samples, while the 7-point standard was used to demonstrate the dynamic range and linearity of the assay. The original stock solutions were made into small aliquots and stored at −80°C to prevent repeated freeze and thaw. 

The nuclear DNA standards were similarly prepared. The nuclear primer sequences are listed in Table 1. A 2.7 kb nuclear fragment containing the calicin gene was amplified from RWPE-1. Calicin is a single-copy nuclear gene that encodes for a basic protein of the sperm head cytoskeleton. 

### 2.6. Quantification of mtDNA Damage and Copy Number Using the Absolute ss-qPCR Method

The amount of relaxed/damaged mtDNA and total copy number were measured by quantifying the original and preheated DNA templates, respectively. The nuclear DNA marker calicin was quantified using the original templates. The qPCR was performed using the Applied Biosystems7500 Fast Real-Time PCR System (ABI) with Power SYBR Fast Green PCR MASTER MIX (ABI) [35]. The original DNA templates and preheated DNA templates and standards were analyzed in triplicates on the same plate. The two-step PCR amplification program for both nuclear DNA and mtDNA was 95.0°C for 30 sec, followed by 40 cycles of 95.0°C for 3 sec and 60.0°C for 30 sec. A melt curve analysis was enabled at the end of amplification. The primer sequences are listed in Table 1. The absolute copy numbers of CO2, D-loop, and calicin were calculated based on the standard curves. Since calicin is a single copy nuclear gene, the cell number could be calculated with the following equation with the assumption that the nuclear equivalent is representative of the cell number (Figure 1):

(2) Cell  number  or  nuclear  equivalent  =Calicin  copy  number  ploidy  of  cell.

The exact copies of damaged and total mtDNA per cell were calculated from:

(3) $$\begin{array}{c} \frac{\text{mtDNAcopies}}{\text{cell}}=\frac{\text{CO2}\;\text{or}\;\text{D-Loop}\;\text{copy}\;\text{number}}{{\text{cell}\;\text{number}}^{*}}, \end{array}$$

*cell number and nuclear equivalent will be used interchangeably from this point.

### 2.7. Data Analysis

All statistical analyses were performed with the aid of Graphpad Prism version 4 software. Unless specified otherwise, the data was analyzed with one-way ANOVA with Dunnett post test, and a *P* < 0.05 is considered significant.

## 3. Results

### 3.1. A New Strategy for the Absolute Quantification of Total and Damaged mtDNA

We have devised a new approach for the absolute quantification of mtDNA structural damage and total copy number in a single analysis. The protocol, illustrated in Figure 1, was comprised of four main steps. The first step consisted in the construction of mtDNA and nuclear DNA standards (Figure 1(a)). Two to three kb DNA fragments containing mtDNA (CO2 or D-loop) and nuclear DNA (calicin) were amplified by PCR from a normal prostate cell line, RWPE-1. The concentration (copies/*μ*L) of these long DNA fragments were quantified and calculated according to (1). The second step was to prepare the DNA templates for qPCR analysis (Figure 1(b)). Each DNA template was split into two equal halves. One half was used for the quantification of relaxed mtDNA and calicin nuclear DNA copies. The other half was pretreated at 95°C for 6 min to unfold any structure and was used for quantifying total mtDNA. The third step consisted in the absolute quantification using qPCR (Figure 1(c)). To obtain mtDNA content per cell, the exact amount of mtDNA and nuclear DNA copies were quantified and calculated from the standard curves according to the equation: copies = “10^(Ct−*b*)/*a*^,” where the cell number was derived from (2). The final step was the interpretation of the data (Figure 1(d)). With this approach, the amount of damaged mtDNA copies/cell, total mtDNA copies/cell, and baseline mtDNA damage (ratio of damaged mtDNA/total mtDNA) were quantified simultaneously.

### 3.2. Dynamic Range, Linearity, Specificity, and Reproducibility of DNA Markers

**Figure 2:**
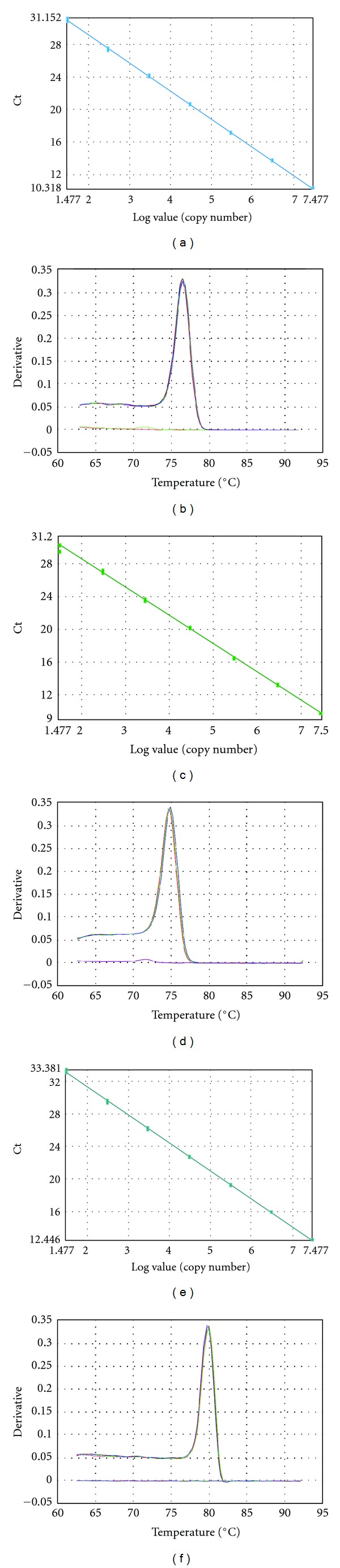
Dynamic range, linearity, specificity, and reproducibility. QPCR amplification of 7-point standard curves from 3 × 10^7^ to 30 copies of (a) CO2, (c) D-loop, and (e) calicin markers. Melting curves showing the negative first derivative of the fluorescence signal of (b) CO2, (d) D-loop, and (f) calicin PCR product.

The standard curve and the melting curve for CO2, D-loop, and calicin amplification were evaluated (Figure 2). Each data point was run in triplicates. The threshold cycles (Ct) of CO2, D-loop, and calicin amplification were based on 10X serially diluted standards ranging from 3 × 10^7^ to 30 copies and were shown in Figures 2(a), 2(c), and 2(e). The PCR amplification efficiencies were 95.8%, 95.7%, and 95.4% for CO2, D-loop, and calicin, respectively. Linearity of the standard curve amplification was maintained in the dynamic range of 10 to 10^7^ since the *R*-values of linear regression lines were 0.9998 for CO2, 0.9988 for D-loop, and 0.9996 for calicin. A single uniform melting peak at 76°C, 75°C, and 79°C was observed for CO2, D-loop, and calicin, respectively, demonstrating the high specificity of the primers (Figures 2(b), 2(d), and 2(f)). The intra-assay reproducibility of the standard was analyzed by calculating the coefficient of variation (CV) of the triplicates. The intra-assay median CV were 0.27%, 0.17%, and 0.12% for CO2, D-loop, and calicin, respectively (Table 2). The interassay CV was calculated with data from two or more independent experiments: the CV were 0.33%, 0.10%, and 0.62% for CO2, D-loop, and calicin, respectively (Table 2). These low CV values demonstrated very high intra- and interassay reproducibility of these new DNA markers.

### 3.3. Total Absolute Quantification of mtDNA Content and Baseline mtDNA Damage in Lymphocytes and Prostate Cancer Cells

**Figure 3:**
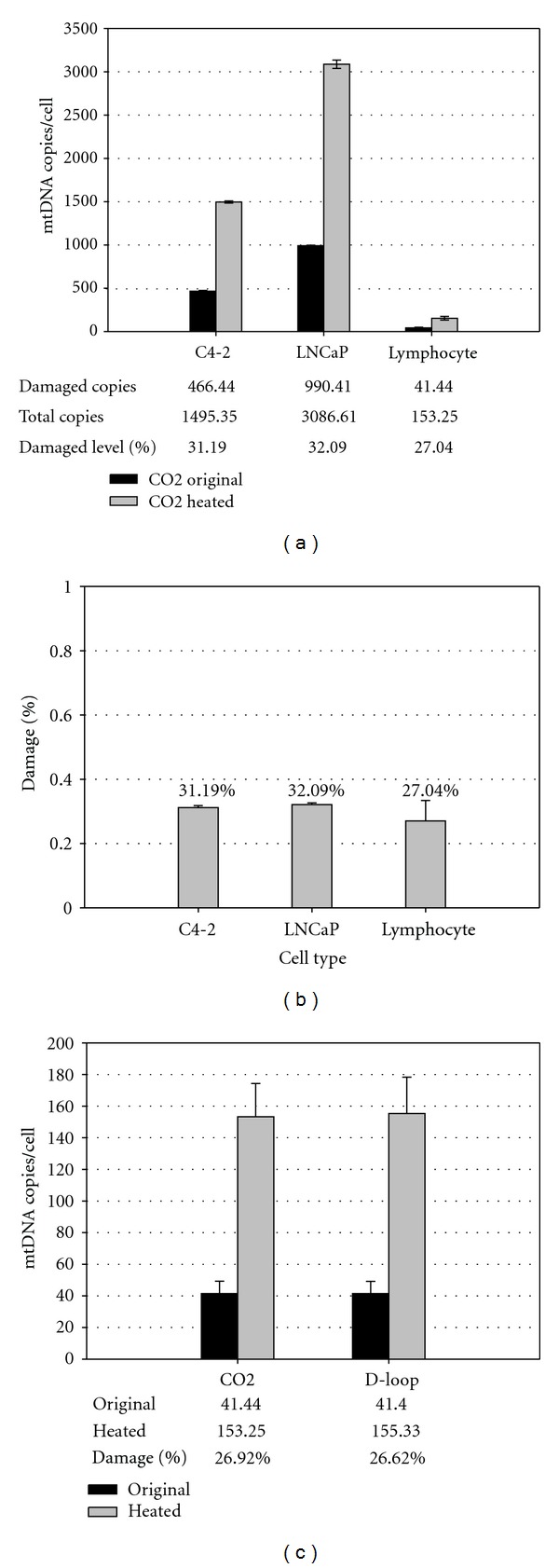
Absolute quantification of total mtDNA and baseline mtDNA damage in lymphocytes and prostate cancer cells. C4-2 (*n* = 2), LNCaP (*n* = 2), and lymphocytes (*n* = 4) were analyzed by ss-qPCR for total mtDNA content, damaged mtDNA number, and level of baseline damage. The cell number was calculated from the copy numbers of calicin, a single copy nuclear marker. (a) With mtDNA CO2 marker, the original (CO2 original) and preheated (CO2-heated) DNA templates were quantified for damaged copies and total mtDNA copies, respectively. (b) The baseline damage level was obtained by dividing damaged copies over total copies. (c) Comparison of CO2 versus D-loop markers in lymphocyte samples (*n* = 4).

Lymphocyte samples and two isogenic prostate cancer cell lines, LNCaP and C4-2, were analyzed for mtDNA content and baseline damage. The prostate cancer cell lines served a reference in method development because the ss-qPCR method was previously developed with these cell lines [6, 31, 35]. In lymphocytes, the total mtDNA content was quantified at an average of 153.25 ± 21.02 copies/cell from 4 individual samples, among which the amount of damaged mtDNA molecules was averaged at 41.44 ± 7.87 copies/cell (Figure 3(a)). In comparison, significantly higher mtDNA contents were detected in prostate cancer cells C4-2 (1495.35 ± 12.45, *P* < 0.01) and LNCaP (3086.61 ± 48.27, *P* < 0.01). The damaged mtDNA copies were 466.44 ± 8.64 and 990.41 ± 6.77 copies/cell, respectively. The baseline damage was calculated with the ratio of damaged mtDNA over total mtDNA: 27.04% of the total mtDNA content was damaged for lymphocytes versus 31.19% and 32.09% for C4-2 and LNCaP, respectively (Figure 3(b)). This assay was highly reproducible; the median intra- and interassay CV were 0.74% and 1.20% for cell lines and 1.87% and 2.33% for lymphocytes, respectively (Table 3). Furthermore, the use of different mtDNA markers, CO2 and D-loop, generated near identical results in terms of total mtDNA content, damaged mtDNA, and baseline damage detected (Figure 3(c)). Indeed, the average CV value obtained between CO2 and D-loop markers was calculated at 0.51%. Thus, these two mtDNA markers were highly consistent and interchangeable in quantitative mtDNA analyses. It was interesting to note that the absolute number of damaged mtDNA molecules was proportionally higher in samples with increased total copy numbers (Figure 3(a)). As such, the ratio between damaged and total copy numbers was a better indicator of the baseline level of DNA damage in a cell (Figure 3(b)). Taken together, the new quantification platform developed in this study provided a highly reproducible method for simultaneous analysis of absolute mtDNA copy number, damaged molecules, and baseline damage in both isolated lymphocytes and cancer cell lines.

### 3.4. *Ex Vivo* mtDNA Damage Responses to Exogenous H_2_O_2_ in Isolated Lymphocytes and in Whole Blood

**Figure 4:**
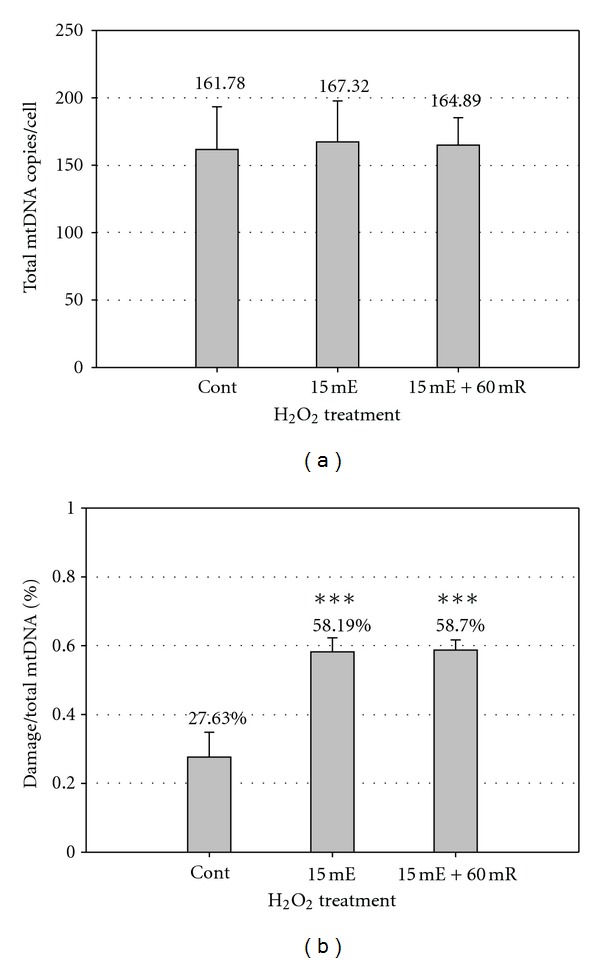
*Ex vivo* mtDNA damage response to exogenous H_2_O_2_ in isolated lymphocytes and whole blood. Experiments were performed to detect the baseline damage level, induced damage by H_2_O_2_ treatment, and repair capacity of white blood cells from healthy volunteers. Lymphocytes were isolated from fresh blood of three volunteers and stored at −80°C. The lymphocytes were split into three groups: untreated control (*n* = 9), 15 min of exposure to 120 *μ*M H_2_O_2_ (*n* = 9), and 15 min of exposure + 60 min recovery (*n* = 6). (a) Total mtDNA content of lymphocytes. (b) MtDNA damage response of lymphocyte samples. (***P < 0.001).

Isolated lymphocytes from 9 healthy men were treated with 0 or 120 *μ*M H_2_O_2_ for 15 min to evaluate induced mtDNA damage and repair activity after 60 min of recovery. The average mtDNA copy number of the untreated control samples was 161.78 ± 31.67 copies/cell (Figure 4(a)). The total mtDNA copy number was not affected by H_2_O_2_ treatment and remained stable across all treatment groups (Figure 4(a)). However, rapid mtDNA damage response was observed. The average baseline damage of untreated control samples was 27.63% (Figure 4(b)). Upon H_2_O_2_ exposure, the fraction of damaged mtDNA increased to 58.19% in lymphocytes, representing a 110.6% increase in induced damage from the control (*P* < 0.001). Interestingly, the induced damage was not repaired after 60 min of recovery, suggesting a lack of repair activity during the recovery period.

**Figure 5:**
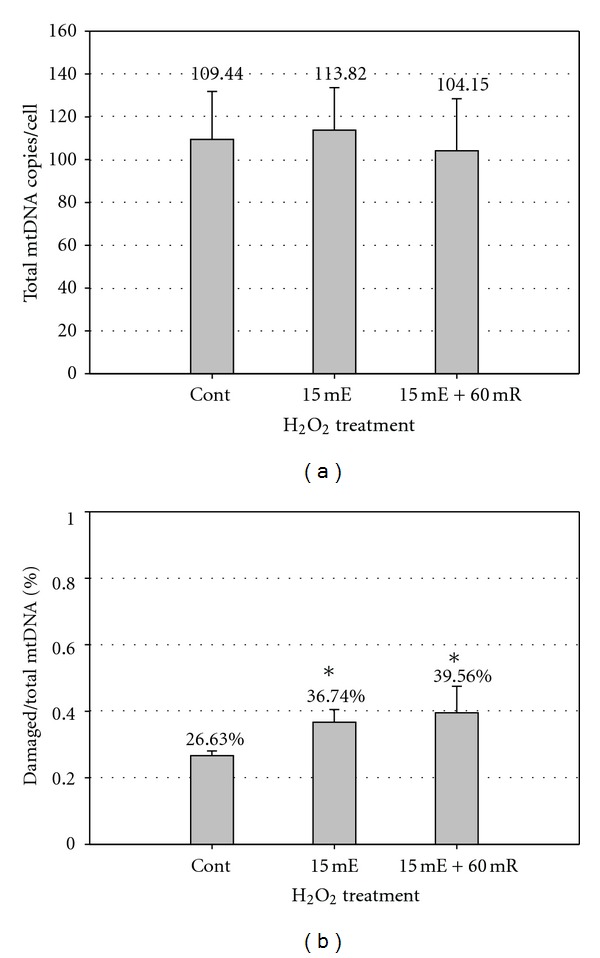
*Ex vivo* mtDNA damage response to exogenous H_2_O_2_ whole blood. Blood from six healthy male volunteers were collected and stored at −80°C. Blood cells from the whole blood samples were separated into three groups in the same manner as lymphocyte samples in Figure 4. (a) Total mtDNA content of whole blood. (b) MtDNA damage response of whole blood. (*P < 0.05).

 As an alternative to the isolated lymphocytes, a small amount of whole blood samples (<1 mL each) from four healthy subjects was tested using the same procedure. The average total mtDNA content of untreated control was 109.4 ± 22.40 copies/cell (Figure 5(a)). Similar to lymphocytes, the average baseline mtDNA damage of the untreated control samples was 26.6% (Figure 5(b)). When treated with 120 *μ*M H_2_O_2_, the damaged fraction of mtDNA increased to 36.7%, representing a 38.0% increase in induced damage as compared to the baseline levels (*P* < 0.05), while the total mtDNA content remained the same (Figure 5(b)). An absence of repair activity was also observed within 60 min recovery after the H_2_O_2_ treatment. However, the whole blood samples had slightly lower mtDNA content and less pronounced mtDNA damage responses as compared to the lymphocytes. This could be caused by the complexity of different types of white blood cells present in whole blood samples. Despite this difference, the overall stress response pattern was similar between the isolated lymphocytes and the whole blood. Thus, the latter may serve as a convenient alternative to isolated lymphocytes in the analysis of mtDNA stress responses in circulating blood.

## 4. Discussion

Based on our previously developed ss-qPCR method [6, 31, 35], we propose a quantitative approach for precise and rapid detection of mtDNA structural damage and copy number change in isolated lymphocytes in a single analysis. We have demonstrated that the new approach had a wide dynamic range and was highly specific and reproducible. A relatively low mtDNA content and baseline level of damage were observed in lymphocytes of healthy men, and the lymphocytes were shown for the first time to exhibit a significant increase in mtDNA damage, followed by little repair activity after 1 h of recovery in an *ex vivo* challenge experiment with H_2_O_2_. This lack of repair activity to H_2_O_2_-induced damage after 1 h of recovery is consistent with a study from Collins et al. in which nuclear DNA repair activity was only observed after several hours (>2 h) [34]. Moreover, we showed that 1 mL of whole blood may serve as a convenient alternative to the isolated lymphocytes in the mtDNA analysis. Thus, mtDNA in blood may be explored as a sensitive surrogate to systemic oxidative stress by simultaneous analysis of multiple end-points in a single test. 

The absolute quantification system developed in this study provides a standard method for the reliable quantification of the precise mtDNA copy number in lymphocytes and whole blood cells. This is achieved through well-defined mtDNA and single-copy nuclear DNA markers and by taking into account the DNA structural effects on qPCR amplification [31]. The relatively low mtDNA copy number revealed in isolated lymphocytes is consistent with very limited data reported in the literature. For example, one study detected *∼*87 to 579 copies/cell with a different real-time PCR method [36] and the other *∼*70 to 320 copies/cell with competitive PCR in lymphocytes [37]. Many studies report mtDNA content on a relative scale [19, 38–41]. However, the relative analysis is limited by the difficulty of comparing results from one study to another and by the significant variations observed in mtDNA content between individuals [42]. To account for the inhibitory effect of the supercoiled DNA structure on qPCR amplification, we have taken steps to ensure an accurate measurement by disrupting the supercoiled mtDNA conformation with a preheating step prior to qPCR analysis. This step is necessary for precise quantification of mtDNA content but has largely been ignored in previous reports. Depending on the manufacturers of the qPCR machinery and chemistry kits, there are wide variations in the duration of the initial hot-activation step for hot-start DNA polymerases, which varies from 10 min to as short as 20 sec (e.g., ABI 7500 Fast System). We have shown that shorter denaturation time at 95°C was insufficient to disrupt all the supercoiled mtDNA conformation in a time- and dose-dependent experiment in prostate cancer cell lines [31]. Therefore, the inclusion of a preheating step in the template preparation is crucial for the accurate mtDNA measurement. In addition to measuring the total mtDNA content, our new system also provides a novel approach for direct quantification of the absolute copies of damaged mtDNA with qPCR. This is in contrast to mtDNA conformational study based on gel electrophoresis coupled with Southern Blot, which requires tedious post-PCR manipulations and is semiquantitative in nature. On the other hand, popular assays such as the comet test for detecting nuclear DNA strand breaks are not applicable to mtDNA due to its small size [34]. The quantification of structural mtDNA damage reported in this study mainly reflects the damage caused by single- and double-stranded breaks, as it was shown that other type of DNA damage such as base lesions or abasic sites had little, if any, effect on the structure [31]. It is interesting to note that the amount of damaged mtDNA changes with the total mtDNA content in a cell. The direct comparison of the damaged mtDNA molecules from different individuals can be compounded by variations in the total content. To normalize this variation, we propose to calculate mtDNA damage based on the percentage of damaged versus total mtDNA molecules in a cell; this ratio of damage is relatively stable and more informative for comparative studies [43]. Moreover, the ratio of damage can be used to infer the baseline or endogenous damage in the isolated lymphocytes or whole blood from the untreated samples; it also quantifies induced mtDNA damage in isolated lymphocytes or a small amount of whole blood cells under oxidative stress. The ability of our approach to measure both endogenous and induced damage/repair responses in *ex vivo* treatments may be used to explore the state of oxidative defence and/or repair capability of individuals with different disease conditions. Indeed, previous studies have suggested that there is an association between systemic oxidative stress and diseases, such as an association of the high prostate cancer risk with severe damage response and poor repair capacity of nuclear DNA in lymphocytes [14].

In conclusion, we have developed an absolute quantification system for rapid measurement of mtDNA structural damage, copy number change, and damage response in isolated lymphocytes and whole blood cells. Systemic oxidative stress is associated with diverse pathological conditions, ranging from neurodegenerative diseases to many types of cancers. It is conceivable that the blood analysis with the multiple mtDNA end-points proposed in the this study may provide a simple and sensitive test to interrogate the nature and extent of systemic oxidative stress for a broad spectrum of clinical investigations, especially when coupled with other established tests such as cell-free circulating mtDNA, the comet assays targeting the nuclear DNA, and the detection of 8-oxo-G base lesions.

## Figures and Tables

**Table 1:** Primer sequences.

Primers	Forward 5′–3′	Reverse 5′–3
CO2 3285 bp long fragment	CCTAGGGTTTATCGTGTGAG	CTAGTTAATTGGAAGTTAACGG
D-loop 2467 bp long fragment	CGCACGGACTACAACCACGAC	CTGTGGGGGGTGTCTTTGGGG
Calicin 2658 bp long fragment	ATTCCAGAAGCCTTTAACTAG	ACAAATGAGACACAAACTACCG
CO2 (for real-time PCR)	CCCCACATTAGGCTTAAAAACAGAT	TATACCCCCGGTCGTGTAGCGGT
D-loop (for real-time PCR)	TATCTTTTGGCGGTATGCACTTTTAACAGT	TGATGAGATTAGTAGTATGGGAGTGG
Calicin (for real-time PCR)	CTGGTCGCTACATCTACATCTC	CAGGTCAGGCAACTTGGTC

**Table 2:** Intra- and interassay CV for standards.

Standards	Intra-assay CV (%)	Interassay CV (%)
CO2	0.27 (0.10–0.72)	0.33 (0.11–0.80)
D-loop	0.18 (0.03–0.16)	0.10 (0.05–0.78)
calicin	0.12 (0.02–0. 67)	0.62 (0.09–1.74)

Median CV value with CV range.

**Table 3:** Intra- and interassay CV for samples.

Sample type	Intra-assay CV (%)	Interassay CV (%)
Prostate cell lines*	0.74 (0.26–1.86)	1.20 (0.68–1.85)
Lymphocytes	1.87 (1.18–2.51)	2.33 (1.45–4.66)

Median CV value with CV range

*RWPE-1, C4-2, LNCaP, PC-3.

## Abbreviations

mtDNA: Mitochondrial DNA
qPCR: Quantitative real-time polymerase chain reaction
8-oxo-G: 8-oxoguanine
ss-qPCR: Supercoiling-sensitive real-time PCR
SSB: Single-strand breaks
DSB: Double-strand breaks.
